# miRNA in Machine-Learning-Based Diagnostics of Oral Cancer

**DOI:** 10.3390/biomedicines12102404

**Published:** 2024-10-21

**Authors:** Xinghang Li, Valentina L. Kouznetsova, Igor F. Tsigelny

**Affiliations:** 1 IUL Scientific Program, La Jolla, CA 92038, USAvkouznetsova@ucsd.edu (V.L.K.); 2 San Diego Supercomputer Center, University of California San Diego, La Jolla, CA 92093, USA; 3 Department of Neurosciences, University of California San Diego, La Jolla, CA 92093, USA

**Keywords:** miRNA, machine learning, oral cancer, diagnostics

## Abstract

Background: MicroRNAs (miRNAs) are crucial regulators of gene expression, playing significant roles in various cellular processes, including cancer pathogenesis. Traditional cancer diagnostic methods, such as biopsies and histopathological analyses, while effective, are invasive, costly, and require specialized skills. With the rising global incidence of cancer, there is a pressing need for more accessible and less invasive diagnostic alternatives. Objective: This research investigates the potential of machine-learning (ML) models based on miRNA attributes as non-invasive diagnostic tools for oral cancer. Methods and Tools: We utilized a comprehensive methodological framework involving the generation of miRNA attributes, including sequence characteristics, target gene associations, and cancer-specific signaling pathways. Results: The miRNAs were classified using various ML algorithms, with the BayesNet classifier demonstrating superior performance, achieving an accuracy of 95% and an area under receiver operating characteristic curve (AUC) of 0.98 during cross-validation. The model’s effectiveness was further validated using independent datasets, confirming its potential clinical utility. Discussion: Our findings highlight the promise of miRNA-based ML models in enhancing early cancer detection, reducing healthcare burdens, and potentially saving lives. Conclusions: This study paves the way for future research into miRNA biomarkers, offering a scalable and adaptable diagnostic approach for various cancers.

## 1. Introduction

MicroRNAs (miRNAs) are small, noncoding RNAs critical in regulating gene expression at the post-transcriptional level, influencing key cellular processes such as development, differentiation, apoptosis, and proliferation [1]. Their role extends into the realm of disease pathogenesis, particularly in cancer, where the dysregulation of miRNA expression has been identified as a significant factor [2]. Traditional approaches to cancer diagnosis, including biopsies and histopathological analyses, are invasive, costly, and demand specialized interpretive skills [3]. These methods are becoming increasingly unsustainable given the rising global incidence of cancer, highlighting the urgent need for alternative diagnostic strategies that are efficient, less invasive, and more accessible [3].

This challenge is magnified by the increasing global incidence of cancer, highlighting the urgent need for non-invasive sample collection and cost-effective diagnostic alternatives [4]. The integration of artificial intelligence (AI) and computational methodologies offers a promising solution [5]. With the exponential growth in genomic and transcriptomics data facilitated by advancements in sequencing technologies, computational prediction of miRNA–disease associations, particularly in the context of oral cancer, has become increasingly feasible [6,7]. Studies like Jiang’s et al., which described a machine-learning (ML) model to predict miRNA targets and their disease associations [8], and Lee’s et al., which used deep-learning (DL) algorithms to analyze miRNA expression profiles for early cancer detection [5], demonstrate the potential of AI to uncover the complex relationships between miRNAs and various cancers, facilitating the discovery of novel biomarkers [9]. Additionally, recent research by Aravind with colleagues on using ML and miRNA for the diagnosis of esophageal cancer [10] and by Kumar with co-authors on Parkinson’s disease diagnosis using miRNA biomarkers and DL [11] further substantiates the efficacy of these approaches in various diseases.

The primary objective of this research project is to explore the potential of miRNA-based ML models as diagnostic tools for oral cancer, a prevalent and often fatal disease [12]. Through the computational analysis of miRNA–disease associations, this study aims to uncover specific miRNAs that could serve as reliable biomarkers for oral cancer [12]. This endeavor promises to enhance our scientific understanding of miRNA functions within the context of cancer and significantly impact society by improving cancer care. By enabling early detection and more informed treatment planning, the project seeks to improve patient prognoses, reduce the healthcare burden of cancer, and ultimately, save lives. Through these efforts, we aim to contribute substantially to the ongoing fight against cancer, offering hope for more effective, less invasive diagnostic methods soon.

## 2. Methods

### 2.1. Data Pre-Processing and Analysis

Our investigation aimed to illuminate the diagnostic potential of miRNAs as non-invasive biomarkers for oral cancer. We identified the liquid biopsy miRNAs linked to oral cancer from open source [12] and utilized computational models (see details below) to predict miRNA–disease associations. Our analysis included two distinct sets of miRNAs: one associated with oral cancer [12], and another consisting of randomly selected miRNAs from the miRBase database [13], which are not associated with oral cancer. The random set of miRNAs selected was distinct from oral cancer-related one.

### 2.2. Generation of miRNA Attributes

The generation of miRNA attributes was structured in three phases: analyzing sequence-based characteristics, identifying potential target genes, and evaluating involvement in cancer-specific signaling pathways [14]. We developed a custom algorithm using miRBase, v. 22.1, to extract and analyze characteristics such as nucleotides composition, frequency, molecular weight, hydrogen bond count, and the presence of specific motifs in the miRNA sequences [15]. This analysis was grounded on the hypothesis that these sequence compositions are crucial for miRNA’s binding ability to target mRNAs, influencing gene regulation [16]. Additionally, the miRDB, v. 6.0, was utilized to identify high-potential target genes for the miRNAs [17], and a Python script was created to efficiently elucidate proper targets of related miRNAs. The extract from the more than 500 gene targets set is presented in Table 1. We also explored the role of miRNAs in cancer by identifying experimentally validated interactions between miRNA–target genes and signaling pathways using the DAVID software, v. 2023q4, specifically pathways significantly associated with cancer [18]. The pathways identified through this comprehensive analysis include key signaling cascades and cellular processes involved in cancer progression and regulation (Table 2). Notably, pathways such as the mTOR signaling pathway, MAPK signaling pathway, TGF-beta signaling pathway, and p53 signaling pathway are known to play critical roles in tumorigenesis and cancer progression. Additionally, the Ras signaling pathway, Hippo signaling pathway, JAK–STAT signaling pathway, and NF-kappa B signaling pathway were identified, highlighting their importance in cell proliferation, apoptosis, and immune response modulation. Pathways like endocrine and other factor-regulated calcium reabsorption, insulin resistance, and choline metabolism in cancer emphasize the metabolic alterations often seen in cancer cells. Furthermore, pathways involved in viral infections, such as Epstein–Barr virus infection and human immunodeficiency virus 1 infection, underline the complex interplay between viral oncogenesis and miRNA regulation.

### 2.3. Software and Database Utilization

Throughout our research, we utilized several key software tools and databases to process and analyze data effectively. miRBase served as a primary resource for obtaining miRNA sequences and related data. miRDB was crucial for identifying and predicting potential target genes for the miRNAs [17]. The Waikato Environment for Knowledge Analysis (WEKA), v. 3.8.6, provided a robust platform for ML analysis, enabling us to apply various algorithms and perform attribute selection [19]. Additionally, the Database for Annotation, Visualization, and Integrated Discovery (DAVID) was instrumental in annotating and integrating biological data, generating the additional signaling pathways descriptors, and further enhancing our understanding of miRNA functions and interactions [18].

### 2.4. Attribute Selection and Machine-Learning Analysis

With a detailed set of attributes encompassing sequence characteristics, target gene associations, and pathway involvements, we moved to the ML analysis phase. Our objective was to train models capable of clearly distinguishing miRNAs associated with oral cancer from those that are not. Using the WEKA platform, we optimized our attribute selection using the InfoGainAttributeEval feature to identify the most impactful attributes for classification [19].

We evaluated the performance of various classification algorithms, focusing on metrics such as accuracy, precision, and other relevant measures [20]. To ensure the reliability and generalizability of our models, we implemented cross-validation techniques [21].

### 2.5. Validation and Testing

The final phase involved the validation of our optimized machine-learning model using an independent dataset of miRNA expressions from patients with oral cancer, obtained from The Cancer Genome Atlas (TCGA), v. 41.0 [22]. This step was crucial to verify the predictive accuracy and potential clinical applications of our model. Validation was conducted using the trained ML model. We submitted the new data to the trained model and elucidated the accuracy of recognition of this dataset as related to oral cancer. The results of this validation are presented below.

Through this comprehensive methodological framework, our study seeks to elucidate the role of miRNAs in blood as non-invasive biomarkers in the diagnosis of oral cancer, potentially advancing cancer detection and treatment strategies.

## 3. Results

### 3.1. Machine-Learning Models

**Figure 1:**
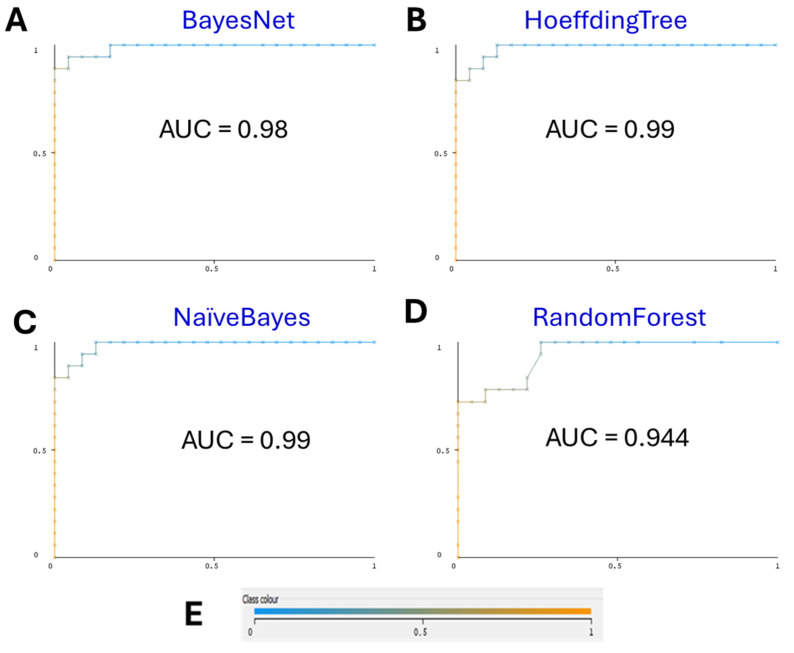
(**A**) BayesNet ROC Curve. The ROC curve for the BayesNet model shows an area under the curve (AUC) of 0.98. This high AUC value indicates excellent model performance in distinguishing between miRNAs associated with oral cancer and those that are not. (**B**) HoeffdingTree ROC Curve. The ROC curve for the HoeffdingTree model demonstrates an AUC of 0.99. While high, it does not match the performance of the BayesNet model in overall accuracy, suggesting less accurate classification. (**C**) Naïve Bayes ROC Curve. The NaïveBayes model’s ROC curve shows an AUC of 0.99. This performance is the same as the HoeffdingTree model, albeit a still lower accuracy. (**D**) RandomForest ROC Curve. The ROC curve for the RandomForest model reveals an AUC of 0.944. This indicates a strong performance but still falls short of the BayesNet model’s AUC. (**E**) Color interpretation of performance curves. Color represents the threshold value set to get the best pair of true FPR/TPR points.

**Figure 2:**
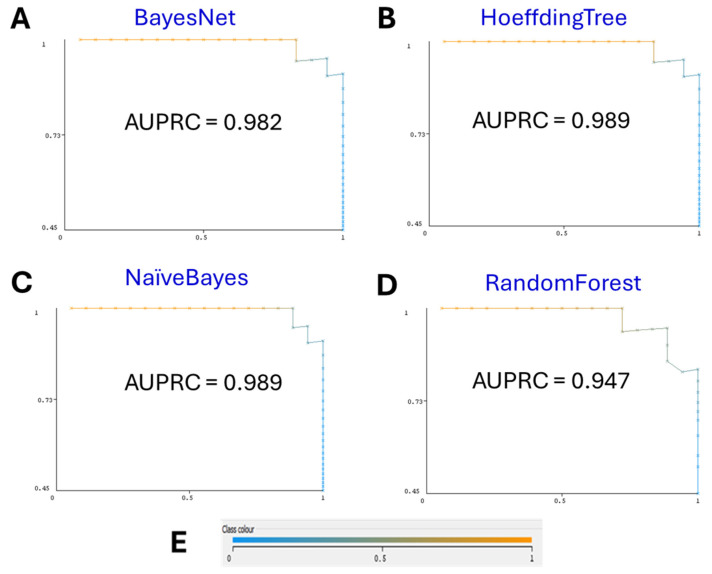
(**A**) BayesNet Precision–Recall (PR) Curve. The PR curve for the BayesNet model shows an area under the curve (AUPRC) of 0.982. This high AUPRC value reflects the model’s excellent precision–recall performance in distinguishing between miRNAs associated with oral cancer and those that are not. (**B**) HoeffdingTree PR curve. The PR curve for the HoeffdingTree model demonstrates an AUPRC of 0.989, slightly outperforming the BayesNet model, indicating superior precision and recall in classification. (**C**) NaïveBayes PR curve. The NaïveBayes model’s PR curve shows an AUPRC of 0.989, matching the performance of the HoeffdingTree model and suggesting highly accurate classification. (**D**) RandomForest PR curve. The PR curve for the RandomForest model reveals an AUPRC of 0.947, indicating strong performance, yet still falling short of the precision–recall accuracy achieved by the BayesNet, HoeffdingTree, and NaïveBayes models. (**E**) Color interpretation of performance curves. Color represents the threshold value set to get the best pair of true FPR/TPR points.

Our final ML model, employing the BayesNet classifier, exhibited superior performance with 5-fold cross-validation, which is supported with performance curves both the receiver operating characteristic (ROC) curve and the precision–recall (PR) curve. The model achieved an accuracy of 95%, precision of 95.4%, recall of 95%, and F-measure of 95%. Notably, the area under the ROC curve (AUC) was 0.98, and the area under the PR curve (AUCPR) was 0.982 (Figure 1 and Figure 2, correspondingly).

These results underscore the model’s efficacy in accurately classifying miRNAs as associated with oral cancer (Table 3). The high precision indicates consistent performance, while the AUC exceeding 0.9 demonstrates the model’s robustness in distinguishing between associated and randomly selected miRNAs [21].

### 3.2. Comparison with Other Algorithms

**Figure 3:**
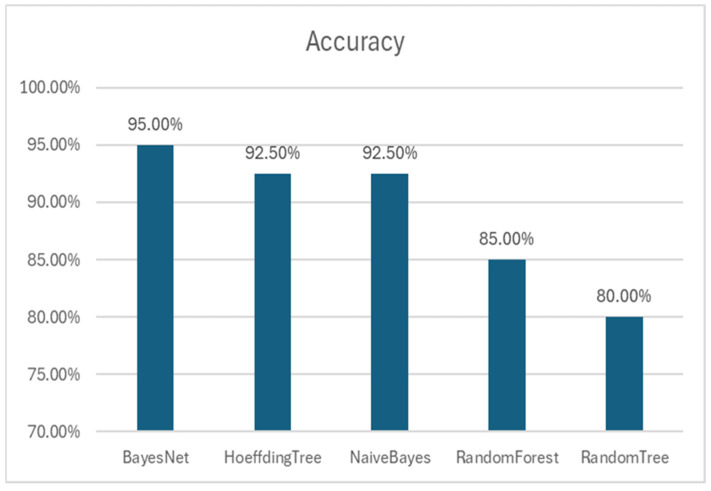
Accuracy of each classifier on the test set with 5-fold cross-validation.

In testing other algorithms, BayesNet consistently outperformed them in terms of key metrics (Figure 3). While other models also showed high performance, the comprehensive evaluation through 5-fold cross-validation confirmed that the BayesNet was the most optimal choice for our objectives [19].

The ROC curves for the various models illustrate the trade-off between sensitivity (true positive rate) and specificity (false positive rate) across different thresholds [23]. The BayesNet model’s ROC curve (Figure 1A) demonstrates the highest area under the curve (AUC = 0.98), indicating superior performance in correctly classifying miRNAs associated with oral cancer. RandomForest shows good performance with various biomarkers [24]. In comparison, the ROC curves for HoeffdingTree, NaïveBayes, and RandomForest models show varying AUC values, signifying similar but slightly worse performance. The high AUC values across all models reflect their strong discriminative abilities, but the BayesNet model stands out as the most robust and reliable when combined with its accuracy.

### 3.3. Optimization of Model Parameters

**Figure 4:**
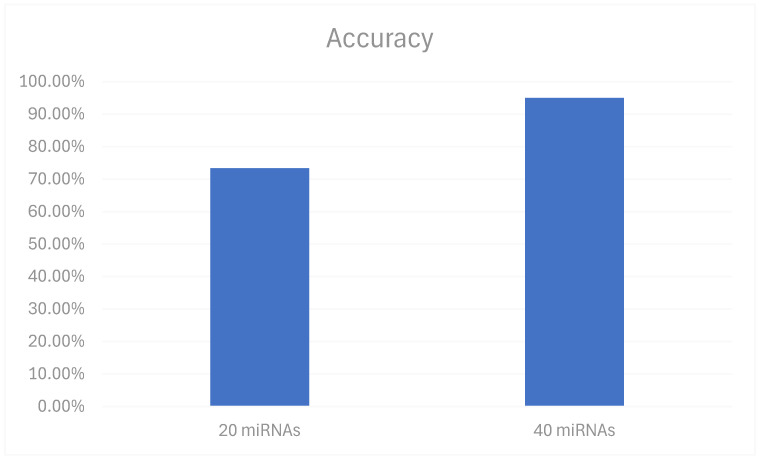
Accuracy of 20 miRNAs and 40 miRNAs for BayesNet classifier.

The BayesNet model’s effectiveness was also influenced by the number of miRNAs used for training and the number of attributes selected for classification. Our analysis confirmed that the model’s performance improved with the increase in the number of miRNAs included in the training set. The model was initially trained with 20 oral cancer-related miRNAs available from the studies [12]. The best results were achieved when the model was trained with a larger dataset that incorporated both the original 20 miRNAs [12] as well as 20 more obtained from additional studies [25]. This expansion led to a noticeable improvement in accuracy (Figure 4), precision, and recall metrics. Specifically, the model achieved an accuracy of 95%, a precision of 95.4%, and a recall of 95%. These enhancements underscore the importance of a comprehensive training dataset in ML models for biological classification tasks. The increased number of miRNAs provided a more diverse set of attributes for the model to learn from, which in turn improved its ability to accurately classify miRNAs associated with oral cancer.

### 3.4. Validation of the Trained Model

Upon validation using independent data, which included miRNAs from patients with oral cancer, the results were the de-identified data that were presented in the TSGA database and all necessary informed consents were obtained from patients before inclusion of their results in the TCGA. The model successfully classified miRNAs as associated with oral cancer, resulting in a classification accuracy of 95%. This high accuracy in an independent test set further validates our model’s utility in real-world diagnostic settings [22]. During this phase, we also tested the robustness and generalizability of the model across different classifiers.

**Figure 5:**
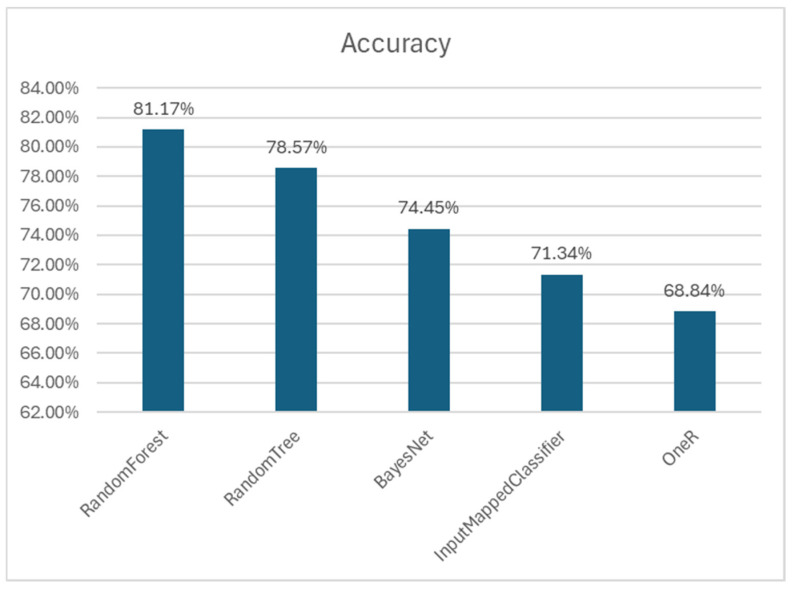
Accuracy of each model on the independent dataset.

The performance accuracies of the best classifiers are shown in Figure 5. The Random Forest classifier showed the highest accuracy at 81.17%, benefiting from its ensemble method which reduces overfitting and enhances generalization by averaging multiple decision trees. The Random Tree classifier followed with an accuracy of 78.57%, demonstrating its straightforward approach to classification but with potential overfitting in some cases. The BayesNet classifier, while achieving a slightly lower accuracy of 74.45%, stood out for its probabilistic framework which makes local decisions based on sufficient statistics, providing it with a distinct advantage in managing noisy and high-dimensional data.

The superior performance of the Random Forest classifier underscores its robustness and effectiveness in miRNA classification tasks. However, the consistent performance of the BayesNet classifier across training, validation, and independent testing phases also highlights its reliability in accurately classifying miRNAs as associated with cancer. This comprehensive validation across these three classifiers reinforces the robustness of our approach and highlights the potential of integrating different machine-learning methods for enhanced miRNA–disease association studies.

### 3.5. Analysis of Model Attributes

The model attributes that contributed most significantly to classification were evaluated using InfoGainAttributeEval. This analysis highlighted the attributes that effectively reduced overall information entropy in the classification process, emphasizing their critical role in distinguishing cancer-associated miRNAs [26].

Our findings elucidated specific pathways and miRNA gene targets associated with cancer. Key genes identified include *AKT1*, *MAPK1*, *PTEN*, *TP53*, and *CDKN1A*. These genes are involved in several well-known cancer-related pathways, such as the PI3K–Akt signaling pathway, the MAPK signaling pathway, and the p53 signaling pathway.

For instance, the PI3K–Akt signaling pathway, which includes the *AKT1* gene, plays a pivotal role in cell proliferation and survival, often being deregulated in cancer. Similarly, the MAPK signaling pathway, represented by the *MAPK1* gene, is integral to cell differentiation and division, with abnormalities often leading to tumorigenesis. The p53 signaling pathway, involving the *TP53* gene, is crucial for regulating the cell cycle and maintaining genomic stability, making it a key player in preventing cancer development.

These insights into the biological characteristics of miRNAs not only enhance our understanding of their involvement in oral cancer but also provide a solid foundation for future diagnostic and therapeutic research. By pinpointing the specific gene targets and pathways that are more prevalent in cancer-associated miRNAs, we can better target these areas for potential treatments and diagnostic tools, advancing the fight against oral cancer.

## 4. Discussion

Existing methods for oral cancer diagnostics include tissue biopsies, imaging techniques like MRI and CT scans, and endoscopic examinations. Tissue biopsies, although accurate, are invasive and can be painful for patients, with accuracy rates varying based on the size and quality of the biopsy sample. Imaging techniques offer non-invasive alternatives but may lack specificity and sensitivity in early-stage detection.

Our approach offers a non-invasive (not requiring a tumor biopsy), highly accurate, and patient-friendly alternative to traditional diagnostic methods. The use of miRNA-based diagnostics reduces the need for painful biopsies and can provide early detection that is both specific and sensitive. Additionally, our method can be more easily deployed in under-resourced settings, where access to advanced imaging and specialized endoscopic equipment is limited.

Our ML model, which leverages the BayesNet classifier [27], demonstrates excellent performance metrics such as high accuracy, area under ROC curve, and precision compared to other classifiers. It is the most effective given the number and types of miRNAs and attributes utilized in its training. The robustness of the BayesNet algorithm is particularly notable in its handling of noisy data. By making local decisions based on sufficient statistics, it minimizes the impact of noise, enhancing the model’s overall classification performance [28]. This ability underscores the potential of our model to accurately distinguish between miRNAs associated with oral cancer and those that are not [29].

The developed ML model’s effectiveness extends beyond its immediate application in identifying oral cancer-associated miRNAs. It possesses the adaptability to be applied to other cancer types or diseases where miRNA associations exist, given the availability of relevant data [30]. This versatility is facilitated by the model’s capacity to classify any miRNA as associated or non-associated with cancer, following a straightforward process of attribute generation and classification via our developed Python script. Additionally, the non-invasive nature of miRNA-based diagnostics presents a significant advantage over traditional methods, making it more accessible and patient friendly.

Looking ahead, expanding the model to include additional attributes like age, gender, and ethnicity—factors known to influence disease incidence and outcomes—could refine its predictive accuracy and applicability [31]. These demographic factors, often linked with varying disease dynamics, could provide deeper insights into the miRNA–disease associations and enhance the model’s contextual relevance. Furthermore, verifying the biological significance of newly identified miRNAs concerning oral cancer through empirical studies could solidify their role in disease pathogenesis. This would not only validate our model’s predictions but also enrich our understanding of miRNA functions in cancer biology [32].

This research has successfully demonstrated the use of an ML model based on miRNA descriptors in diagnostics of oral cancer. By incorporating sequence-based attributes, predicted target genes, and pathway analyses into our model, we have discerned the most salient features for miRNA classification, achieving an accuracy of 86.8% in training phases and 83% in validation against an independent dataset. The implications of these findings are profound, emphasizing the viability of miRNA-based markers as non-invasive diagnostic tools. This approach has the potential to complement traditional diagnostic methods such as endoscopy, particularly in under-resourced settings, thereby broadening the accessibility of cancer diagnostics [33]. Moreover, the pathways and target genes identified in this study open new possibilities for targeted therapeutic interventions, addressing key aspects of cancer proliferation [34].

Our study is distinct in its methodological innovation and the specific combination of computational techniques used to analyze miRNA characteristics [18]. This novel approach not only enhances the accuracy of cancer-associated miRNA identification but also provides a scalable model that could be applied to other cancers and diseases with known miRNA signatures. Moving forward, this groundwork will enable further explorations into less invasive, more accessible diagnostic and therapeutic tools, potentially significantly altering the paradigm of cancer care.

To apply our methods, scientists can download our ML model to their computers and submit sets of miRNAs from new patients. The model will recognize whether these patients have oral cancer. Such results can suggest the next step of diagnostics using more invasive methods. We would be happy to help with the implementation of this method.

## Figures and Tables

**Table 1:** miRNA gene target descriptors.

miRNAs	*Genes*							
hsa-miR-301a-5p	*MAB21L2*	*PPM1L*	*KLHL42*	*STK39*	*TNRC6B*	*SMAD1*	*EPC2*	*ERLIN1*
hsa-miR-3160-5p	*C5orf24*	*ELMOD2*	*RPS6KA6*	*LSM12*	*UBN2*	*FBXO28*	*NLK*	*LOXL2*
hsa-miR-1297	*SLC8A1*	*SERINC5*	*CDK8*	*LARP1*	*NAP1L5*	*CAMSAP1*	*PLEKHH1*	*IQCJ*
hsa-miR-3677-5p	*MBNL2*	*SLC5A3*	*ETNK1*	*LEF1*	*ULK2*	*FAM136A*	*COL10A1*	*ZIC5*
hsa-miR-4535	*RGL1*	*TNFAIP1*	*POLR3G*	*ATP11C*	*BAG4*	*TMC7*	*RSPRY1*	*PARPBP*
hsa-miR-6732-5p	*CDH11*	*DIP2A*	*SLC7A11*	*PEX13*	*BLOC1S2*	*REEP3*	*PFKFB2*	*MSMO1*
hsa-miR-6717-5p	*ESR1*	*STRADB*	*RHOQ*	*E2F7*	*SLC25A36*	*FBXL19*	*ULK1*	*KDM1B*
hsa-miR-3619-5p	*MGA*	*SLC2A13*	*OTUD4*	*NHS*	*MAB21L1*	*PLOD2*	*UBR3*	*ITGB1BP1*
hsa-miR-4746-3p	*ZNF236*	*CIPC*	*NAB1*	*NUS1*	*USP3*	*ZNF608*	*CDK6*	*RC3H1*
hsa-miR-4746-3p	*TADA2B*	*TET2*	*CHORDC1*	*STRBP*	*NAA15*	*CEP350*	*UBE2G1*	*MED19*
hsa-miR-4746-3p	*BNC2*	*CASZ1*	*CREBRF*	*SLC25A16*	*ALDH5A1*	*SULF1*	*CREB1*	*MFN2*
hsa-miR-1304-3p	*DHX9*	*USP9X*	*TET1*	*CLASP2*	*MTM1*	*BRWD1*	*ADM*	*AMMECR1L*
hsa-miR-570-3p	*NR2C2*	*FAM98A*	*B3GNT5*	*TET3*	*GNA13*	*ZSWIM6*	*SLC45A4*	*ZFAND3*
hsa-miR-21-3p	*ZFHX4*	*TP53INP1*	*PHF3*	*PITPNC1*	*SYT10*	*EIF2S1*	*BBX*	*MAPK1*
hsa-miR-32-3p	*TSC22D2*	*PTEN*	*KCNJ2*	*OSBPL11*	*GSK3B*	*FRMD4B*	*MTDH*	*NEO1*
hsa-miR-4763-3p	*CTNND2*	*KLHL42*	*STK39*	*TNRC6B*	*SMAD1*	*EPC2*	*ERLIN1*	*DOLPP1*
hsa-miR-6796-3p	*PPM1L*	*RPS6KA6*	*LSM12*	*UBN2*	*FBXO28*	*NLK*	*LOXL2*	*CBL*
hsa-miR-486-3p	*ELMOD2*	*CDK8*	*LARP1*	*NAP1L5*	*CAMSAP1*	*PLEKHH1*	*IQCJ*	*SEC24C*
hsa-miR-3605-3p	*SERINC5*	*ETNK1*	*LEF1*	*ULK2*	*FAM136A*	*COL10A1*	*ZIC5*	*DMXL2*
hsa-miR-548ae-3p	*SLC5A3*	*POLR3G*	*ATP11C*	*BAG4*	*TMC7*	*RSPRY1*	*PARPBP*	*C17orf49*
hsa-miR-525-3p	*EIF5A2*	*FAM160B1*	*COL19A1*	*SLC30A7*	*HOOK3*	*NR3C1*	*HPSE2*	*C17orf49*
hsa-let-7a-3p	*TMTC2*	*C21orf91*	*44991*	*PLEKHM3*	*KIAA0232*	*KIAA1217*	*BAZ2A*	*PIM1*

**Table 2:** miRNA gene target-related pathways.

Pathways	
Endocrine and other factor-regulated calcium reabsorption	Oxytocin signaling pathway
mTOR signaling pathway	Adrenergic signaling in cardiomyocytes
Prostate cancer	Progesterone-mediated oocyte maturation
Insulin resistance	MAPK signaling pathway
AMPK signaling pathway	MicroRNAs in cancer
Autophagy—animal	TGF-beta signaling pathway
Breast cancer	Oocyte meiosis
Endometrial cancer	Signaling pathways regulating pluripotency of stem cells
Hepatocellular carcinoma	Hippo signaling pathway
Kaposi sarcoma-associated herpesvirus infection	JAK–STAT signaling pathway
Human cytomegalovirus infection	Endocytosis
Longevity regulating pathway	Measles
Choline metabolism in cancer	Alcoholic liver disease
Phospholipase D signaling pathway	Hepatitis B
African trypanosomiasis	Tuberculosis
Allograft rejection	Pathogenic Escherichia coli infection
Type I diabetes mellitus	Epstein–Barr virus infection
Pathways in cancer	Human immunodeficiency virus 1 infection
Pertussis	Lipid and atherosclerosis
Leishmaniasis	B cell receptor signaling pathway
Chagas disease	C-type lectin receptor signaling pathway
Toll-like receptor signaling pathway	T cell receptor signaling pathway
Ras signaling pathway	Proteoglycans in cancer
Melanoma	Long-term depression
p53 signaling pathway	Fc epsilon RI signaling pathway
NF-kappa B signaling pathway	Gastric cancer
Toxoplasmosis	Melanogenesis

**Table 3:** Performance metrics of the top five classifiers.

Classifier	Accuracy	Precision	Recall	F-Measure	AUC	AUPRC
BayesNet	0.950	0.954	0.950	0.950	0.980	0.982
HoeffdingTree	0.925	0.934	0.925	0.924	0.990	0.989
NaïveBayes	0.925	0.934	0.925	0.924	0.990	0.989
RandomForest	0.850	0.852	0.850	0.849	0.944	0.947
RandomTree	0.850	0.800	0.800	0.800	0.798	0.738

## Data Availability

Data are contained within the article. A code is available upon request.
